# Challenges for Medical Students in Applying Ethical Principles to Allocate Life-Saving Medical Devices During the COVID-19 Pandemic: Content Analysis

**DOI:** 10.2196/52711

**Published:** 2024-01-05

**Authors:** Hsing-yen Hsieh, Chyi-her Lin, Ruyi Huang, Guan-chun Lin, Jhen-Yu Lin, Clydie Aldana

**Affiliations:** 1 School of Medicine for International Students College of Medicine I-Shou University Kaohsiung Taiwan; 2 Department of Pediatrics E-Da Hospital Kaohsiung Taiwan; 3 Holistic Medicine Department of Family and Community Medicine E-Da Hospital Kaohsiung Taiwan; 4 Data Science Degree Program National Taiwan University and Academia Sinica Taipei Taiwan; 5 Division of Family Medicine, Taipei Tzu Chi Hospital, Buddhist Tzu Chi Medical Foundation and School of Medicine, Tzu Chi University Hualien Taiwan

**Keywords:** virtual patient, virtual patients, medical resources distribution, medical ethical education, COVID-19 pandemic, ethics, medical student, medical students, medical ethics, decision-making, ethical dilemna, simulation, reasoning, decision support, medical guideline, medical guidelines, medical devices, medical device, life-saving, thematic analysis, virtual platform

## Abstract

**Background:**

The emergence of the COVID-19 pandemic has posed a significant ethical dilemma in the allocation of scarce, life-saving medical equipment to critically ill patients. It remains uncertain whether medical students are equipped to navigate this complex ethical process.

**Objective:**

This study aimed to assess the ability and confidence of medical students to apply principles of medical ethics in allocating critical medical devices through the scenario of virtual patients.

**Methods:**

The study recruited third- and fourth-year medical students during clinical rotation. We facilitated interactions between medical students and virtual patients experiencing respiratory failure due to COVID-19 infection. We assessed the students’ ability to ethically allocate life-saving resources. Subsequently, we analyzed their written reports using thematic analysis to identify the ethical principles guiding their decision-making.

**Results:**

We enrolled a cohort of 67 out of 71 medical students with a mean age of 34 (SD 4.7) years, 60% (n=40) of whom were female students. The principle of justice was cited by 73% (n=49) of students while analyzing this scenario. A majority of them expressed hesitancy in determining which patient should receive life-saving resources, with 46% (n=31) citing the principle of nonmaleficence, 31% (n=21) advocating for a first-come-first-served approach, and 25% (n=17) emphasizing respect for patient autonomy as key influencers in their decisions. Notably, medical students exhibited a lack of confidence in making ethical decisions concerning the distribution of medical resources. A minority, comprising 12% (n=8), proposed the exploration of legal alternatives, while 4% (n=3) suggested medical guidelines and collective decision-making as potential substitutes for individual ethical choices to alleviate the stress associated with personal decision-making.

**Conclusions:**

This study highlights the importance of improving ethical reasoning under time constraints using virtual platforms. More than 70% of medical students identified justice as the predominant principle in allocating limited medical resources to critically ill patients. However, they exhibited a lack of confidence in making ethical determinations and leaned toward principles such as nonmaleficence, patient autonomy, adherence to legal and medical standards, and collective decision-making to mitigate the pressure associated with such decisions.

## Introduction

The COVID-19 pandemic has caused millions of deaths and countless hospitalizations worldwide owing to critical conditions caused by the virus [1]. This has raised the ethical dilemma of allocating scarce life-saving devices to critically ill patients [2-5].

Physicians often make clinical decisions based on scientific evidence to avoid moral distress [3,6,7]. However, clinical decisions may have to be made under time constraints. Preparing physicians to apply appropriate ethical principles, have self-confidence in making choices, and prevent moral trauma has become essential during the pandemic [8].

The principles of autonomy, justice, beneficence, and nonmaleficence commonly serve as guiding references for allocating scarce medical resources [9]. However, these principles have multiple interpretations when facing limited resources and can be based on utilitarianism, egalitarianism, or deontology [10]. Utilitarianism believes that the primary obligation is not to treat people equally, but to maximize the greatest amount of happiness for the greatest number of people; the best actions would be based on what brings the best benefit. By contrast, egalitarianism upholds the rights and interests of individuals, which should be equally protected [10]. Deontology judges the morality of choices by its conformity with a moral norm [11], regardless of its consequences. Persad et al [12] present a comprehensive framework for the allocation of scarce medical resources grounded in the core principles of autonomy, justice, beneficence, and nonmaleficence. Their framework encompasses 4 distinct ethical value categories, including equal treatment, prioritization of the most vulnerable, maximizing overall benefits, and recognition of social usefulness. Within each category, 2 competing ethical principles emerge, yielding a total of 8 subprinciples that provide detailed guidance aligned with the overarching ethical values [12]. The core values or principles that medical students prefer or overlook when facing ethical dilemmas are unclear and require further study.

The School of Medicine for International Students at I-Shou University has a 4-year Doctor of Medicine program that collaborates with the Ministry of Foreign Affairs and enrolls college graduates from countries with official diplomatic ties to Taiwan. Due to the limited medical resources in such students’ home countries, they may face the challenge of a shortage of life-saving medical facilities in clinical practice. Therefore, equipping them with the knowledge and skills to allocate life-saving medical devices to critically ill patients, based on reasonable principles of medical ethics, is crucial. The use of virtual patients for teaching medical humanities may strengthen the effectiveness of medical ethics education [13,14]. Considering the challenges imposed by the COVID-19 pandemic, this solution aims to offer a secure and personalized training environment, transcending the boundaries of time and space. By doing so, students can become fully engrossed in virtual scenarios, enriching their learning experiences.

The objective of this study was to assess the ability and confidence of medical students to apply principles of medical ethics in allocating critical medical devices through the scenario of virtual patients.

## Methods

### Study Design

We designed a virtual scenario and asked medical students to allocate lifesaving medical devices to only 1 patient. In this scenario, a 62-year-old COVID-19-infected patient with respiratory failure was admitted to the intensive care unit. Medical students were instructed to interview a virtual patient and review the patient’s laboratory and imaging findings. They then were asked to make clinical diagnoses and adopt appropriate ethical principles to determine whether to remove the extracorporeal membrane oxygenation (ECMO) device from an 80-year-old patient currently using it and reallocate it to the new younger patient. After making their decision, the students were requested to write a short essay addressing the ethical conflicts they encountered in making the choice.

### Ethical Considerations

We explained the rationale for this qualitative study and recruited third- and fourth-year medical students from the School of Medicine for International Students Program when they undertook clinical rotation at the hospital. All participants completed the virtual clinical scenarios within 4 hours in May 2021, during the COVID-19 pandemic in Taiwan, after signing an informed consent form. This study was approved by the E-Da Hospital Institutional Review Board (no. EMRP05109N and EMRP04111N), and the data were not identifiable. The teaching and evaluation of students were not affected by whether they participated in the research.

### Case Scenario

Leona is a 62-year-old retired woman. She had been well without any underlying disease until recently being diagnosed with COVID-19 pneumonitis. Her lung condition continuously deteriorated, and ECMO was the last resort to support her tissue oxygenation. However, the only available ECMO machine was currently being used by an 80-year-old patient with multiple chronic illnesses who remained unstable after receiving ECMO treatment, with minimal chances of recovery.

The students were given the above scenario to assess and answer relevant questions. One of the questions was “Will you continue to let the 80-year-old patient use the ECMO, or let Leona use the ECMO instead? Please explain your decision and your reasons to support it.”

The medical students could use the 4 principles of medical ethics or base their responses on their individual analytical perspectives and reasoning for the allocation of limited medical resources.

### Data Analysis

Age (>25 vs ≤25 years) and sex (male vs female) served as basic demographic variables, with the age of 25 years as a threshold of maturity. Grade (third vs fourth year) represented differences in clinical exposure experiences [15]. Textual content analysis was performed by 2 of the authors to search for keywords and summarize the students’ responses independently. The keywords were encoded and categorized for both quantitative and qualitative analyses. We used the principles of summative content analysis, which combines the quantitative counting of specific content or words or terms with latent content analysis to identify and categorize their meanings. In brief, we created a new coding category for any newly introduced terms in the assignment, and then assessed conceptual similarities to determine whether to further organize these codes into additional categories with appropriate names.

The qualitative analysis consisted of the following steps:

1. The coding items included the final decision of the students (for whom to use), which core medical ethical principles were applied with various degrees in their choices, and whether viewpoints other than ethics, such as medical guidelines or legislation, were mentioned.
2. The reasons for the students’ final decisions were classified according to the patient they selected, either the 62-year-old younger patient or the 80-year-old patient with multiple comorbidities. Our analysis focused on encoding the ethical justifications provided by the medical students to support their final decisions. We omitted considerations related to their alternative choices during the decision-making process.
3. The classification of reasoning for those who made a decision was primarily based on the students’ understanding and interpretations in their essays, which Persad et al [12] mentioned were equality, vulnerability, maximizing the quality of life, and contribution to society. The original resource allocation principles were designed for the distribution of medical supplies among a group of individuals. However, the present case pertains to the treatment decision for an individual patient, further complicated by the fact that one patient had already been put on a ventilator. By contextualizing the principles within the framework of the present case, we eliminated the applicability of 4 subprinciples: lottery, saving the most lives, reciprocity, and giving priority to the worst off (ie, sickest first).
4. If students displayed reluctance in making a choice, we also coded their explanations for the perception that ethical decision-making might not be suitable, categorizing these explanations as “undetermined” or “both unqualified.”
5. The main reasons for the students’ final decisions were classified into medical, legal, and ethical perspectives.
6. The coding process was independently judged by 2 researchers with expertise in qualitative research. Any inconsistencies in coding were resolved by reviewing the classification descriptions to refine the precision of category definitions and revisiting the context to ensure accurate coding.

## Results

### Student Demographics

From 2021 to 2022, a total of 71 international third- and fourth-year clinical medical students who were facing the COVID-19 pandemic most significantly were enrolled. Of these, 67 students (33 third-year and 34 fourth-year students) from 12 countries participated in the study. Because 4 fourth-year medical students did not participate, the response rate was 94%. Overall, 40 (60%) participants were female and 61 (91%) were older than 25 years. Most medical students were from the Kingdom of Eswatini, accounting for 48% (n=32) of the total group (Table 1 and Multimedia Appendix 1).

**Table 1:** Basic information of the students.

Demographic			Medical students (n=67), n (%)
**Sex**			
	Male	27 (40)	
	Female	40 (60)	
**Age (years)**			
	>25	61 (91)	
	≤25	6 (9)	
**Seniority year**			
	Third	33 (49)	
	Fourth	34 (51)	
**Country of origin**			
	The Kingdom of Eswatini	32 (48)	
	Saint Lucia	7 (10)	
	Belize	7 (10)	
	Kiribati	5 (7)	
	Honduras	3 (4)	
	The Marshall Islands	3 (4)	
	Saint Kitts and Nevis	3 (4)	
	Paraguay	2 (3)	
	Saint Vincent & The Grenadines	2 (3)	
	Palau	1 (1)	
	Haiti	1 (1)	
	Solomon Islands	1 (1)	

### Choosing the Best Candidate for ECMO Allocation

Of the 67 participating students, age group (<25 vs ≥25 years old), sex (male vs female), and seniority year (third vs fourth year) did not affect patient selection preferences, and a larger proportion of students from Eswatini (21/32, 66%) selected the 80-year-old patient for ECMO compared to the rest of the students (39/67, 58%). The majority of students decided to continue treating the 80-year-old patient with ECMO (Table 2).

Additionally, 5 (8%) students argued that the medical information provided was not sufficient to make decisions that were highly dependent on factors such as the patient’s condition, the course of the disease, and legal requirements. One student (1%) suggested that, in accordance with medical guidelines, neither patient met the conditions to be a candidate for ECMO. A possible reason for them to abstain from decision-making could be the pressure they experienced while facing an ethical dilemma. As one student (no. 16) stated:

Doctors should not take the treatment away of one person and give it to another, regardless of the odds of survival rate of these two patients, because it means that we are taking the role of God, deciding who lives and who dies.

Another student (no. 20) stated:

I don't believe I have the right to decide who is more deserving or who needs this equipment more.

**Table 2:** Choosing the most suitable patient for extracorporeal membrane oxygenation treatment.

Patient selected	Students (n=67), n (%)
80-year-old	39 (58)
62-year-old	22 (33)
Undetermined	5 (8)
Both unqualified	1 (1)

### Students’ Perspective of Allocating Limited Resources

Building upon the framework proposed by Persad et al [12], this study identified 4 coding categories after excluding subprinciples that were deemed inapplicable to the current case. In accordance with the students’ final decisions regarding the most suitable recipient for ECMO, we categorized the reasons endorsed by the students (Table 3). The primary justifications for selecting an 80-year-old patient included nonmaleficence (n=31, 46%), first-come-first-served (n=21, 31%), and patient autonomy (n=17, 25%). Students grounded their decisions in 3 of the 4 ethical principles, arguing that in this particular scenario, those advocating for the principle of nonmaleficence contended that physicians lacked the authority to withdraw a life-saving device in active use. “First-come-first-served” represents 1 of the 4 interpretive angles of the justice principle from Persad’s framework. Students believed that the life of each patient held equal value, and those who received treatment first should be allowed to continue treatment. Students who mentioned patient autonomy were particularly concerned about the absence of informed consent and its potential legal implications for health care providers.

The reasons for selecting the 62-year-old patient primarily revolved around the principle of justice. The utilitarian principle of maximum benefit was the most popular: 31% (n=21) of students mentioned that medical resources should be reserved for patients who can survive the longest and have the best quality of life. When comparing who had better survival probabilities, some students suggested that medical guidelines should serve as the basis for the final decision. Overall, 10% (n=7) of students made decisions depending on who had contributed more to society as a whole, and 4% (n=3) prioritized the disadvantaged, where the disadvantaged can be interpreted as the younger patient.

Students who expressed an “undetermined” stance believed that decision-making authority should be entrusted to guidelines, which could be either principles collectively established by physicians within the hospital (n=4, 6%), hospital policies (n=4, 6%), local laws (n=4, 6%), or decisions made by the hospital’s ethics committee (n=3, 4%). Alternatively, some advocated for decisions to be made collectively by physicians within the hospital (n=1, 1%), by the patients’ families (n=1, 1%), or based on other information relevant to the patient’s condition (n=1, 1%). One student expressed a “both unqualified” position and approached the issue from a medical rather than an ethical perspective. The student asserted that, based on the guidelines, neither of the 2 patients met the criteria for usage.

**Table 3:** Multiple-choice analysis of the reasoning for case selection among students.

Reasoning for selected patient		Students (n=67), n (%)
**80-year-old**		
	Nonmaleficence (physician has no right to withdraw)	31 (46)
	Treat patients equally (first come, first served)	21 (31)
	Patient’s autonomy (law issue)	17 (25)
	Withdraw can’t prove 62-year-old patient’s survival	2 (3)
**62-year-old**		
	Higher survival rate, save the maximum quality of life (medical issue)	21 (31)
	Rewarding social usefulness	7 (10)
	Giving priority to the worst off; youngest first	3 (4)
**Undetermined**		
	Decided by medical guidelines, collective decision	4 (6)
	Decided by hospital	4 (6)
	Depend on law	4 (6)
	Decided by the ethics committee	3 (4)
	Decided by 80-years-old patient’s family member	1 (1)
	Depend on other medical information	1 (1)
**Both unqualified**		
	Both are unqualified for ECMO^a^ per guidelines	1 (1)

^a^ECMO: extracorporeal membrane oxygenation.

### Adequacy of Using Medical Ethical Principles

In total, 73% (n=49) of students cited the principle of justice while analyzing this case. When ethical principles were in conflict, the principle of justice was most commonly cited. The frequencies of ethical principles considered by medical students in making final decisions (coding as simple choice) were as follows: 48% (n=32) used the principle of justice, 25% (n=18) used the principle of nonmaleficence, 12% (n=8) used the principle of patient autonomy, and 9% (n=6) were unable to provide a definitive response.

### Confidence in Ethical Decision-Making

Overall, 75% (n=50) of the participants analyzed the case from other perspectives, such as medicine and law, and 25% (n=18) made their final decision based on the principles mentioned in the clinical guidelines. These students were more inclined toward the scientific mode of thinking, believing that evidence-based medicine is objective and may provide clear standards that can give them a sense of security. Students no. 23 and 31, respectively, indicated the following:

I can respond to this situation based on scientific evidence.

A comprehensive assessment of the pathology of the patient’s current condition and the state of illness is a major consideration in decision-making.

For 12% (n=8) of the medical students, their final decisions were made from a legal perspective; that is, they stated that the decision should be made in accordance with the law of the state. They emphasized that physicians should protect themselves from being sued and provide decision-making authority to the patient or family. The patients or their family members should sign the emergency consent form, allowing the patient or family to participate in decision-making. As stated by student no. 40:

Medical care providers must consider medical laws, including those for removing the machine from the patient and withholding services from patients.

Additionally, 6% (n=4) of the medical students believed that medical institutions should provide clear guidelines or set up ethics committees to make collective decisions, thus preventing individual doctors from facing the pressure of decision-making. Student no. 18 stated:

I will follow the organization's code of ethics. The handling rules approved by a specific organization that will guide you in such situations so that you do not face a violation of the law.

## Discussion

### Principal Findings

ECMO is recommended for severe COVID-19-related acute respiratory distress syndrome to reduce mortality [16]. Currently, there is no evidence-based ethical guidance for prioritizing ECMO when resources are limited during the COVID-19 pandemic [17]. Justice is the preferred principle in virtual settings, although students have diverse interpretations. Nearly half of the students used additional principles, such as nonmaleficence and respect for patient autonomy, to prevent further harm while making ethical decisions. Multiple perspectives were adopted by three-fourths of the students.

The context of clinical situations is important for making clinical decisions based on ethical dilemmas [18]. The use of virtual patients for medical education may strengthen the effectiveness of medical ethics education [13,14]. Using virtual patients for clinical decision-making training among international medical students offers several advantages [19-21]. It provides a safe training environment amidst the COVID-19 pandemic and allows for diverse case presentations from multiple countries and cultures [22]. The application of virtual care has flourished internationally during the post-COVID era. The Cleveland Medical Center in the United States has also explored the integration of remote and virtual health care. Medical institutions in the southern United States have proved that virtual diagnosis and treatment can alleviate caregiver burden and promote patient care [23]. Our study has provided evidence that combining virtual training with ethical reasoning in solving ethical dilemmas may present a safe environment for learning clinical decision-making and offer opportunities for improvement.

Students were asked to think about and answer questions according to the situation of the virtual patient. More than half of the students chose the oldest or the sickest patient to be the best candidate. The clinical scenario that was tested involved ex-post triage, which entails discontinuing ongoing treatment in favor of a newly arrived patient. Particularly in the context of a pandemic with limited resources (eg, ventilators), the primary objective is to maximize overall benefits for all individuals. While challenging, medical physicians may need to make the difficult decision of reallocating life-saving facilities from the most critically ill patients to those who have a higher probability of survival [5]. During a pandemic, rationing may require the withdrawal of care in order to provide ventilators to patients who are given higher priority, a reason foreign to many front-line clinicians [24]. Sharing and leveraging the diverse responses of medical students themselves can serve as a valuable reference for fostering innovative approaches in medical ethics education and facilitating ethical deliberation on challenging medical issues.

Medical students must define problems, identify potential solutions, and also inform patients about the current treatment options. The students’ understanding of patient autonomy and informed consent was superficial and formalistic; they were more concerned about obtaining consent or documents to avoid legal proceedings. Recent discussions on the principles of patient autonomy have concluded that superficial autonomy cannot guarantee patient autonomy [25-27]. Moreover, physicians should make more efforts to meet the best interests of patients [28,29]. Considering students’ diverse backgrounds, it is important to take into account their various learning styles to enhance and personalize educational materials [30].

The inability to establish a definitive ethical guideline capable of resolving issues stemming from the scarcity of medical resources underscores the complexity of the situation. Furthermore, factors such as patients possessing varying medical needs, financial capabilities to cover medical expenses, and the policies of health care institutions can all impact the ethical judgments of students [31,32]. Therefore, teachers can take the opportunity to emphasize to students that the premise of patient autonomy and informed consent is to uphold the patient’s right to live, and promoting the well-being of the patient is the core value of the principle of patient autonomy. To ensure the patient’s autonomy is respected, physicians should make decisions that benefit the patient’s overall health and care.

Students were unfamiliar with philosophical and ethical reasoning and were under pressure to make ethical decisions about allocating life-saving medical modalities. They tended to analyze ethical issues from both medical and legal perspectives [33,34]. Most medical students relied on objective medical guidelines, legal documents, or hospital management systems to help them make decisions while lacking life-saving medical modalities. Experts might erroneously assume that by dutifully adhering to the code’s regulations they fulfill all pertinent ethical obligations. Similarly, many people hold the belief that by fulfilling all applicable legal prerequisites, they have fulfilled their moral duties. It is important to note that what may be deemed ethically correct does not always find support within the confines of the law. Legal education places emphasis on the introduction of statutes and their applicability, while ethical education delves into the reasoning process underlying diverse ethical decisions. Within medical ethics education, an exploration of students’ abilities to discern the implications of various ethical decisions and make informed value judgments is paramount [35]. Some students believe that developing medical guidelines can serve as a substitute for individual ethical decision-making. Use of the specification method to solve ethical dilemma questions has limitations. If a specification eliminates contingent conflict, it may be arbitrary, lack impartiality, or fail for other reasons. We cannot avoid judgements that balance different principles or rules in the very act of specifying them. It also seems pointless or unduly complicated to engage in specification in many circumstances [35].

To foster the development of medical students’ ethical thinking, it becomes crucial to provide them with opportunities to analyze cases using established ethical frameworks with proper guidance [5]. Furthermore, facilitating the sharing of diverse perspectives on case analysis can also prove valuable in nurturing community-specific morality, which draws its foundations from culture, religion, and institutional systems [35]. Based on our study, we proposed that the necessity of strengthening medical ethics education stems from the following: acknowledging physicians’ needs for independent ethical decisions during a pandemic, recognizing the irreplaceability of clinical ethical judgment over legal rules and medical guidelines, elevating students’ ethical reasoning abilities, and elucidating the core value and application scope of patient autonomy.

This study explored the current status of critical ethical decision-making from the diverse perspectives of international medical students and provided information using a virtual patient scenario. Heist et al [36], using case summaries, found that 5 sessions of virtual patient case scenarios significantly improved students’ clinical reasoning abilities. In light of the rapid advancement of virtual medical education platforms amidst the COVID-19 pandemic, it is suggested that medical schools proactively integrate a series of diverse virtual patient ethics decision-making exercises. This strategic inclusion aims to foster robust and well-rounded ethical education training for medical students, equipping them with the necessary skills to navigate complex ethical dilemmas in their future medical practice.

Through incorporating the survey in the formal class activity, we received a robust 94% response rate from a diverse group of medical students [37]. However, this study has some limitations. First, the interface and language processing technique of the virtual system could be more user-friendly in mimicking the true clinical interaction with patients. The responses of virtual patients were based on a predetermined script derived from a limited database design, making it difficult to respond to students’ more in-depth or spontaneous questions. Second, owing to the limited number of participants (n=67) and the fixed setting of a single virtual patient, students’ responses may not have been extrapolated. If the current medical resources and institutional policy differ, students might make various decisions.

### Conclusion

This study addressed the need for practical clinical ethics training in medical education by using virtual patients to offer students simulated scenarios for cultivating decision-making experiences. It compiled diverse perspectives from students of various cultural backgrounds, enhancing their capacity for comprehensive ethical considerations. The research suggests a more effective curriculum development approach by combining individual case studies with a collective analysis of answers. As future physicians, these students will benefit from this training when making time-sensitive ethical decisions based on all stakeholders’ viewpoints. This study also identifies a lack of student confidence in making ethical decisions related to patients’ lives. It highlights the need to foster the independent ethical decision-making competency of medical students.

## Appendix

Multimedia Appendix 1 Global distribution of international medical students.

## Abbreviations

ECMO: extracorporeal membrane oxygenation
