# Supplementary figures and images for: Challenges for Medical Students in Applying Ethical Principles to Allocate Life-Saving Medical Devices During the COVID-19 Pandemic: Content Analysis
Source: JMIR Med Educ. 2024 Jan 5;10:e52711. doi: 10.2196/52711 (PMC10799279; doi:10.2196/52711)

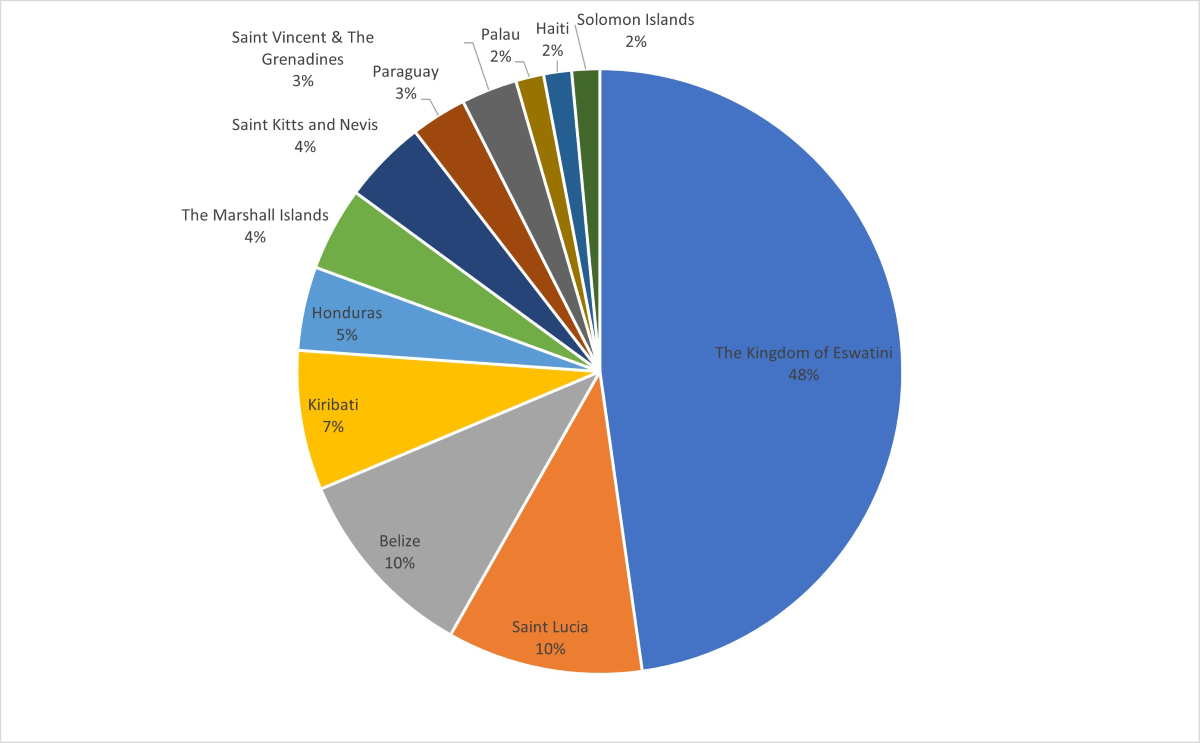

Supplement: Multimedia Appendix 1 [file mededu_v10i1e52711_app1.png]
